# Metabolic versatility of the nitrite-oxidizing bacterium *Nitrospira marina* and its proteomic response to oxygen-limited conditions

**DOI:** 10.1038/s41396-020-00828-3

**Published:** 2020-11-23

**Authors:** Barbara Bayer, Mak A. Saito, Matthew R. McIlvin, Sebastian Lücker, Dawn M. Moran, Thomas S. Lankiewicz, Christopher L. Dupont, Alyson E. Santoro

**Affiliations:** 1 Department of Ecology, Evolution and Marine Biology, University of California, Santa Barbara, CA USA; 2 Marine Chemistry and Geochemistry Department, Woods Hole Oceanographic Institution, Woods Hole, MA USA; 3 Department of Microbiology, IWWR, Radboud University, Nijmegen, The Netherlands; 4 J. Craig Venter Institute, La Jolla, CA USA

**Keywords:** Water microbiology, Microbial biooceanography, Marine microbiology, Bacterial genomics, Bacterial physiology

## Abstract

The genus *Nitrospira* is the most widespread group of nitrite-oxidizing bacteria and thrives in diverse natural and engineered ecosystems*. Nitrospira marina* Nb-295^T^ was isolated from the ocean over 30 years ago; however, its genome has not yet been analyzed. Here, we investigated the metabolic potential of *N. marina* based on its complete genome sequence and performed physiological experiments to test genome-derived hypotheses. Our data confirm that *N. marina* benefits from additions of undefined organic carbon substrates, has adaptations to resist oxidative, osmotic, and UV light-induced stress and low dissolved *p*CO_2_, and requires exogenous vitamin B_12_. In addition, *N. marina* is able to grow chemoorganotrophically on formate, and is thus not an obligate chemolithoautotroph. We further investigated the proteomic response of *N. marina* to low (∼5.6 µM) O_2_ concentrations. The abundance of a potentially more efficient CO_2_-fixing pyruvate:ferredoxin oxidoreductase (POR) complex and a high-affinity *cbb*_*3*_-type terminal oxidase increased under O_2_ limitation, suggesting a role in sustaining nitrite oxidation-driven autotrophy. This putatively more O_2_-sensitive POR complex might be protected from oxidative damage by Cu/Zn-binding superoxide dismutase, which also increased in abundance under low O_2_ conditions. Furthermore, the upregulation of proteins involved in alternative energy metabolisms, including Group 3b [NiFe] hydrogenase and formate dehydrogenase, indicate a high metabolic versatility to survive conditions unfavorable for aerobic nitrite oxidation. In summary, the genome and proteome of the first marine *Nitrospira* isolate identifies adaptations to life in the oxic ocean and provides insights into the metabolic diversity and niche differentiation of NOB in marine environments.

## Introduction

Aerobic nitrite (NO_2_^−^) oxidation is the main biochemical nitrate (NO_3_^−^)-forming reaction, carried out during the second step of nitrification [1]. In marine ecosystems, nitrate is the dominant form of biologically available nitrogen, which is rapidly assimilated by phytoplankton in surface waters and accumulates in the deep sea [2].

Nitrite-oxidizing bacteria (NOB) are chemolithoautotrophic microorganisms found within four known bacterial phyla (Proteobacteria, Nitrospirae, Nitrospinae, and Chloroflexi) [3]. The genus *Nitrospira*, within the Nitrospirae phylum, is the most diverse NOB genus and consists of at least six phylogenetic sublineages [3]. *Nitrospira* are ubiquitously present in natural and engineered ecosystems, including oceans [4, 5], freshwater habitats [6], soils [7, 8], saline-alkaline lakes [9], hot springs [10], wastewater treatment plants [11–13], and aquaculture biofilters [14, 15]. In human-made ecosystems, *Nitrospira* is generally considered to be adapted to low NO_2_^−^ concentrations [16]. In the open ocean, however, where NO_2_^−^ concentrations are exceedingly low, NOB affiliated with the phylum Nitrospinae appear to be the dominant nitrite oxidizers [4], whereas *Nitrospira* are found in relatively high NO_2_^−^ environments including sediments and deep sea hydrothermal vent plumes [17, 18]. *Nitrospira* also dominate over Nitrospinae-affiliated bacteria in some deep-sea trench environments [19, 20]. High NO_2_^−^ concentrations are found coincident with low O_2_ concentrations in oxygen minimum zone (OMZ) waters [21, 22] in a feature known as the secondary nitrite maximum with concentrations reaching ﻿~1–5 µM NO_2_^−^ [23, 24]. Despite the O_2_-dependence of all known NOB, NO_2_^−^ oxidation can still be detected at nanomolar O_2_ concentrations [25].

Some metabolic features appear to be common among *Nitrospira*, based on genomic analyses to date. Immunocytochemical analyses of a representative of *Nitrospira* sublineage I and metagenomic analyses of *Nitrospira defluvii* indicated a periplasmic orientation of nitrite oxidoreductase (NXR) [26, 27], the key enzyme for NO_2_^−^ oxidation, and the presence of the ﻿O_2_-sensitive reductive tricarboxylic acid (rTCA) cycle for inorganic carbon fixation [26]. These results suggest that *Nitrospira* evolved from microaerophilic or anaerobic ancestors [26]. *N. defluvii* lacks genes for classical oxidative stress defense enzymes present in most aerobic organisms including catalase and superoxide dismutase (SOD), indicative of adaptations to low O_2_ environmental niches [26]. However, other *Nitrospira* species encode both types of enzymes [28, 29], suggesting different O_2_ tolerances within members of the *Nitrospira* genus. In addition to NO_2_^−^ oxidation, *Nitrospira* exhibit a high metabolic versatility, growing aerobically on hydrogen [30] or anaerobically on organic acids while respiring nitrate [28]. Recently, the capability for the complete oxidation of ammonia to nitrate (comammox) was identified in representatives of sublineage II *Nitrospira* [14, 31], however, comammox *Nitrospira* appear to be absent in marine systems [31].

*Nitrospira marina* Nb-295^T^, the type species of the genus *Nitrospira*, was isolated from a water sample collected at a depth of 206 m from the Gulf of Maine in the Atlantic Ocean over 30 years ago [5]. It is the only *Nitrospira* species isolated from the oceanic water column and the environmental conditions favorable for *Nitrospira* in the ocean remain largely unexplored. Here, we investigated the metabolic potential of *N. marina* Nb-295^T^ based on its complete genome sequence and compared the proteome signatures of cultures grown under atmospheric O_2_ tension and under low O_2_ conditions typically encountered in OMZs.

## Materials and methods

### Cultivation of *N. marina* Nb-295

A cryopreserved stock of *N. marina* Nb-295^T^ was obtained from the culture collection of John B. Waterbury at the Woods Hole Oceanographic Institution. Strain Nb-295 was grown in 60 mL polycarbonate bottles (Nalgene) in 50 mL autotrophic mineral salts medium at pH 7.8 containing 2 mM NaNO_2_ (Table S1), and bottles were incubated at 25 °C in the dark without agitation. Mixotrophic growth was tested through the individual addition of the following organic carbon substrates to the culture medium of duplicate cultures (final concentrations): 150 mg L^−1^ yeast extract, 150 mg L^−1^ tryptone, 0.5 mM pyruvate, or 1 g L^−1^ glycerol. NO_2_^−^ consumption was measured as previously described [32] and growth was monitored by flow cytometry (see Supplementary Methods). To test for chemoorganotrophic growth, the culture was transferred (2% inoculum) into NO_2_^−^-free medium containing either 1 mM formate or 1 mM pyruvate, and 100 µM ammonium chloride, which served as nitrogen source.

For incubations at different O_2_ concentrations, triplicate cultures of *N. marina* Nb-295 were grown at 22 °C in 400 mL of medium containing 70% natural seawater and 2 mM NaNO_2_ as described by Watson et al. [5] in 500 mL polycarbonate bottles (Nalgene) with a custom-made sparging rig. Bottles were constantly bubbled (flow rate: 15 mL min^−1^) with one of two sterile custom gas mixes containing either 0.5% or 20% oxygen, 300 ppm CO_2_, and a balance of high-purity N_2_. NO_2_^−^ concentrations were measured as a proxy for growth as described above and 10 mL aliquot of each culture was fixed at the last time point (2% formaldehyde, 1 h, 4 °C) for cell enumeration on an epifluorescence microscope as previously described [33].

### DNA extraction, genome sequencing, and annotation

High molecular weight genomic DNA was extracted from stationary phase cultures using a CTAB extraction protocol [34] and sequenced on the PacBio platform at the US Department of Energy Joint Genome Institute (JGI). 754,554 reads were produced, with 209,987 passing quality control. The assembly was conducted using HGAP (v 2.2.0.p1) with improvement with Quiver [35] resulting in a single contig.

Gene annotation was conducted using JGI’s Integrated Microbial Genomes and Microbiomes pipeline [36] and the MicroScope platform [37]. Manual curation included sequence similarity searches using BLASTP (e-value <1e^−30^) [38] against the Transporter Classification database [39] and protein domain searches using InterProScan (release 72.0) [40]. Signal peptides were identified with SignalP 5.0 [41] to determine if proteins were potentially addressed to the membrane and/or released to the periplasmic space. A list of manually curated annotations can be found in Data Set 1.

Phylogenomic analysis was performed using 120 concatenated phylogenetic marker genes from representatives of the phylum Nitrospirae/Nitrospirota as implemented in the Genome Taxonomy Database Toolkit (GTDB-tk) version 1.1.1 [42]. (see Supplementary Methods).

### Protein extraction and proteome analyses

Cells for proteomic analysis were harvested when [NO_2_^−^] dropped to ~500 µM, corresponding to exponential growth of strain Nb-295. Each culture was mixed with an equal volume of a house-made fixative [43] similar to the commercially available solution RNALater (Thermo Fisher), and filtered by vacuum filtration onto 25 mm, 0.2 µm pore size Supor filters (Pall). 200 mL of fixed culture (equivalent to 100 mL of growth medium) were filtered for protein extraction and proteomic analyses. Filters were frozen at −80 °C until extraction. A detailed description of the procedures used for protein extraction and purification can be found in the Supplementary Methods. Global (untargeted) proteomes were analyzed on a Fusion Orbitrap mass spectrometer using one-dimensional nanospray separation and data-dependent acquisition based on Saito et al. [44] (see Supplementary Methods for detailed protocols). Select targeted quantitative proteomic assays using custom-made isotopically-labeled (^15^N) peptide standards were designed and samples were analyzed again by parallel reaction monitoring mass spectrometry using mass spectral information from the global proteome analyses as previously described [44] (also see Supplementary Methods). Tryptic peptides from ten proteins of interest, including nitrite oxidoreductase subunit alpha (NxrA), were targeted for absolute quantitation (see Data Set 2). Cellular NXR concentrations were calculated based on NxrA peptide concentrations using the average carbon content of *N. marina* cells (152 fg cell^−1^, Santoro et al.*, unpublished*) and an estimated cellular protein:carbon ratio of 50% based on experimentally determined values [45]. NXR complex density on the cellular membrane was calculated using an estimated NXR complex size of 63 nm^2^ [27] and an estimated cell surface area of 2.45 µm^2^ (assuming a cylindrical shape with a length of 1.75 µm and a radius of 0.2 µm based on previously determined cell dimensions of *N. marina* [5]).

Differential levels of expression between the two treatments (i.e., atmospheric O_2_ and low O_2_ conditions) were tested with the DESeq2 Bioconductor package (version 1.20.0) [46] in the R software environment (version 3.5.0) [47] using spectral counts as input data as previously described [48]. Proteins with a mean spectral count below 6 across all treatments were excluded from the analysis. In DESeq2, only proteins that increased in abundance under low O_2_ conditions were considered (‘altHyphothesis=greater’), *P* values were adjusted using the Benjamini-Hochberg method (“pAdjustmethod=BH”) and independent filtering was omitted (“independentFiltering=FALSE”). Changes in protein abundance (as determined by spectral counts) were considered statistically significant when adjusted *P* values were lower than or equal to 0.05 (see Data Set 3). While DESeq2 has a high precision and accuracy [49], it is more conservative than other methods on low-count transcripts/proteins [50]. Proteins of interest were visualized with the pheatmap package (version 1.0.12) [51] in the R software environment [47]. The normalized spectral abundance factor was calculated as proxy for relative protein abundances [52], and values were square-root transformed to improve visualization of low abundant proteins.

## Results and discussion

### Genome analysis

**Fig. 1:**
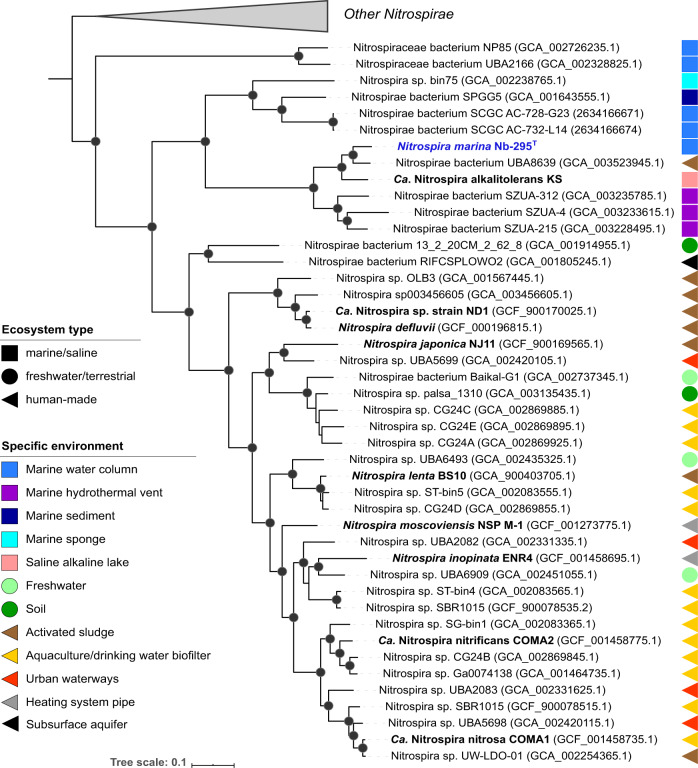
Maximum likelihood phylogenetic tree of representatives of the phylum Nitrospirae. The multiple sequence alignment consisting of 120 concatenated phylogenetic marker genes contained 95 genomes and metagenome-assemble genomes (MAGs) from the Genome Taxonomy Database (GTDB) (Release 04-RS89, 19 June 2019), the genome of *N. marina*, the MAG of *Ca*. N. alkalitolerans and two open ocean single-cell amplified genomes (SAGs), AC-738-G23 and AC-732-L14. All MAGs and SAGs were estimated to be ≥50% complete with ≤5% contamination. Nodes with UFBoot support of at least 95% are indicated as black filled circles. Cultured representatives are shown in bold. The scale bar represents 0.1 substitutions per site.

The genome of *N. marina* Nb-295 is a single element of 4,683,627 bp and contains 4272 coding DNA sequences (CDS) including one rRNA operon. A 5578 bp region of 99.7% identity on each end of the scaffold suggests circularization into a single chromosome. No plasmids or extra-chromosomal elements were identified. The G + C content is 50.04%, lower than in other *Nitrospira* species and the marine nitrite-oxidizers *Nitrospina gracilis* and *Nitrococcus mobilis* (Table S2). Phylogenomic analysis of available closed genomes, metagenome-assembled genomes (MAGs) and single-cell amplified genomes affiliated with the phylum Nitrospirae (see Supplementary Methods) placed *N. marina* Nb-295^T^ within a cluster of genomes derived from marine and saline environments (Fig. 1). The most closely related *Nitrospira* MAG (UBA8639) was obtained from a laboratory-scale nitrification reactor; however, the reactor influent consisted of 33% untreated seawater [53], suggesting a marine origin of this MAG. The 16 S rRNA gene sequence of *N. marina* Nb-295^T^ clustered together with environmental sequences derived from marine sediments and marine aquaculture biofilters (Fig. S1), and shared 99.1% and 97.9% sequence identity with the cultured lineage IV representatives *Nitrospira sp*. Ecomares 2.1 [15] and *Ca*. Nitrospira alkalitolerans [9], respectively.

#### Nitrogen metabolism and respiratory chain

The genome of *N. marina* encodes orthologs of all known proteins required for NO_2_^−^ oxidation, including the putatively periplasm-oriented NXR complex. Three candidates each of genes for NxrA, NxrB, and NxrC, and two additional NxrC-like proteins were identified (Table S2, Data Set 1). The genes *nxrA* and *nxrB*, encoding the alpha and beta subunits of NXR, are colocalized in three clusters, whereas all *nxrC* candidate genes are localized separately from *nxrAB*, as previously described for *Nitrospira moscoviensis* [54]. The NxrA subunits share 87.3–88.9% amino acid identity, the NxrB subunits share 98.8–99.5% amino acid identity, and the putative NxrC subunits are less conserved, sharing between 33.7% and 86.9% amino acid identity.

Like all other analyzed *Nitrospira* genomes, *N. marina* encodes a putative copper-dependent NO-forming nitrite reductase (NirK), yet its function in *Nitrospira* and other NOB remains unknown. *N. marina* also encodes the ferredoxin-dependent nitrite reductase (NirA) for assimilatory nitrite reduction, which appears to be conserved in *Ca*. Nitrospira lenta and *N. defluvii* but absent in other *Nitrospira* [55]. In addition, *N. marina* encodes three high affinity ammonium transporters (Amt) enabling direct uptake of reduced N for assimilation, and cyanate lyase to hydrolyze cyanate to ammonium and CO_2_ (Data Set 1). In contrast to some *Nitrospira* species [13, 28, 31], *N. marina* does not encode a urea transporter or urease, which would catabolize urea to ammonia for N assimilation (Table S2).

Previously sequenced genomes of *Nitrospira* contain multiple copies of several complexes of the respiratory chain [26, 28, 29, 54]. *N. marina* encodes two paralogous copies of complex I, one of which contains a duplication of NADH:quinone oxidoreductase subunit M (NuoM) and lacks genes for NuoE, NuoF and NuoH (Data Set 1), which is a characteristic feature of *Nitrospira* genomes [54, 56]. Furthermore, the *N. marina* genome contains two copies of complex III, two cytochrome *bd* oxidases and seven putative cytochrome *bd*-like oxidases (Data Set 1), which show limited partial similarity to canonical cytochrome *bd* oxidase subunit I as described for *N. moscoviensis* [54]. One of these cytochrome *bd*-like oxidases (*bd*-like_6) contains putative heme b and copper binding sites potentially functioning as a novel terminal oxidase as previously proposed [26]. *N. marina* also encodes for a *cbb*_3_-type terminal oxidase, which usually exhibit high affinities for O_2_ [57]. This feature is shared with the closely related *Ca*. N. alkalitolerans [9] and with the more distantly related marine NOB *Nitrospina gracilis* [58], but absent in all other *Nitrospira* species sequenced thus far. In addition to the canonical H^+^-translocating F_1_F_0_-ATPase (complex V), *N. marina* also encodes a putative alternative Na^+^-translocating N-ATPase (Data Set 1), which potentially contributes to the maintenance of the membrane potential and the generation of a sodium motive force (SMF) as suggested for *Ca*. N. alkalitolerans [9]. Furthermore, a H^+^-translocating pyrophosphatase (H^+^-PPase) with homology to *Leptospira*/protozoan/plant-type enzymes [59] was identified. H^+^-PPases couple the translocation of H^+^ to the hydrolysis of the biosynthetic by-product pyrophosphate (PP_i_), which is suggested to be an adaptation to life under energy limitation [60]. The *N. marina* genome also contains an alternative complex III (ACIII) module, which shares similarity with that from sulfur-reducing Acidobacteria [61]. Like canonical complex III, ACIII also functions as a quinol oxidase transferring electrons to cytochrome *c* and contributes to energy conservation (Refojo et al., 2012). With the exception of the comammox bacterium *Ca*. Nitrospira nitrificans [14], no homologs of ACIII modules were identified in any other NOB genome.

*N. marina* encodes a putative *hyb*-like operon containing ﻿four subunits of a cytoplasmic Group 3b [NiFe] hydrogenase and six accessory proteins involved in hydrogenase assembly and maturation (Data Set 1). This type of hydrogenase appears to be conserved in the marine NOB *Nitrospina gracilis* 3/211 and *Nitrococcus mobilis* Nb-231 [58, 62], *Ca*. N. alkalitolerans [9] and in comammox *Nitrospira* [14, 31] (Table S2). In addition to catalyzing the reversible, NAD^+^-dependent oxidation of hydrogen, these so-called sulfhydrogenases have been reported to reduce elemental sulfur (S^o^) or polysulfides to hydrogen sulfide (H_2_S) [63]. Furthermore, the *N. marina* genome encodes a putative periplasmic sulfite:cytochrome *c* oxidoreductase, which might couple sulfite (SO_3_^2−^) oxidation to sulfate (SO_4_^2−^) with the reduction of cytochrome *c* as previously suggested for *Nitrospina gracilis* [58]. Contrastingly, sulfide/quinone oxidoreductase, which is speculated to mediate sulfide oxidation in *Nitrococcus* [62], is lacking. Whether or not these enzymes are involved in energy conservation using H_2_S and SO_3_^2−^ as alternative substrates in NOB remains to be experimentally validated.

#### Central carbon metabolism

In agreement with other *Nitrospira* genomes [13, 26, 54, 55], *N. marina* encodes the complete gene repertoire for the rTCA cycle for carbon dioxide (CO_2_) fixation, including the key enzymes ATP-citrate lyase and 2-oxoglutarate/pyruvate:ferredoxin oxidoreductase (Data Set 1). In the ocean, inorganic carbon is predominately available in the form of bicarbonate (HCO_3_^−^) and to a much lesser extent as CO_2_. Five inorganic anion transporters (SulP family) with homology to BicA HCO_3_^−^ uptake systems of the cyanobacterium *Synechococcus* [64] were identified in the *N. marina* genome (Data Set 1). Two of these putative BicA-like transporters are colocalized with genes encoding Na^+^/H^+^ antiporters (NhaB family), which could drive the uptake of HCO_3_^−^ via Na^+^ extrusion under alkaline conditions as suggested for the ﻿cyanobacterium *Aphanothece halophytica* [65] and *Ca*. N. alkalitolerans [9]. *N. marina* also encodes one putative SulP-related bicarbonate transporter fused to a carbonic anhydrase and four genes encoding putative alpha, beta and gamma carbonic anhydrases (Data Set 1), which can convert the imported HCO_3_^−^ to CO_2_ for inorganic carbon fixation via the rTCA cycle.

In addition to the rTCA cycle, *N. marina* encodes all required genes for the oxidative TCA cycle for pyruvate oxidation via acetyl-CoA, complete gluconeogenesis and glycolysis pathways, and the oxidative and non-oxidative branches of the pentose phosphate pathway (Data Set 1), which are common features of all sequenced *Nitrospira* genomes [9, 26, 28, 29, 55]. Furthermore, biosynthetic pathways for all amino acids except methionine were identified in the *N. marina* genome. Although *N. marina* encodes a vitamin B_12_-dependent methionine synthase (MetH) (Data Set 1), it appears to lack additional enzymes of known methionine biosynthesis pathways, a trait shared by all other sequenced *Nitrospira* species and *Nitrospina gracilis* [9, 26, 28, 29, 55, 58]. However, as *N. marina* can grow in artificial seawater medium without added methionine, we hypothesize that an alternative unknown pathway for the early steps of methionine biosynthesis functions in *Nitrospira* and *Nitrospina*. The *N. marina* genome contains genes for the biosynthesis and degradation of the storage compounds glycogen and polyphosphate (Data Set 1). In contrast to other *Nitrospira* and the marine NOB *N. gracilis* and *N. mobilis* that encode a glgA-type glycogen synthase, *N. marina* encodes alpha-maltose-1-phosphate synthase (glgM) and alpha-1,4-glucan:maltose-1-phosphate maltosyltransferase (glgE) for the synthesis of glycogen via alpha-maltose-1-phosphate.

#### Use of organic carbon substrates

*N. marina* has been reported to be an obligate chemolithotroph that grows best in medium supplemented with low concentrations of organic compounds including pyruvate, glycerol, yeast extract and peptone [5]. Thus, we investigated the genomic basis for this observation and conducted additional physiological experiments.

In addition to its complete glycolysis pathway and oxidative TCA cycle, a putative carbohydrate degradation operon was identified, consisting of a sugar ABC transporter module, beta-glucosidase, a putatively secreted glycoside hydrolase (GH15) and a carbohydrate-binding protein (Data Set 1). *N. marina* also encodes two putative carbohydrate-selective porins (OprB), a sugar:sodium symporter (SSS family), a putative galactonate/glucarate transporter (MFS superfamily) and a putative carboxylate transporter (DASS family). The genomic repertoire for the catabolic degradation and assimilation of peptides and amino acids, including transporter proteins for di- and oligopeptides (ABC and POT/PTR families), multiple amino acid:cation symporters (SSS, DAACS and AGCS families), amino acid/polyamine transporters (APC superfamily) and multiple putatively secreted peptidases are present (Data Set 1).

**Fig. 2:**
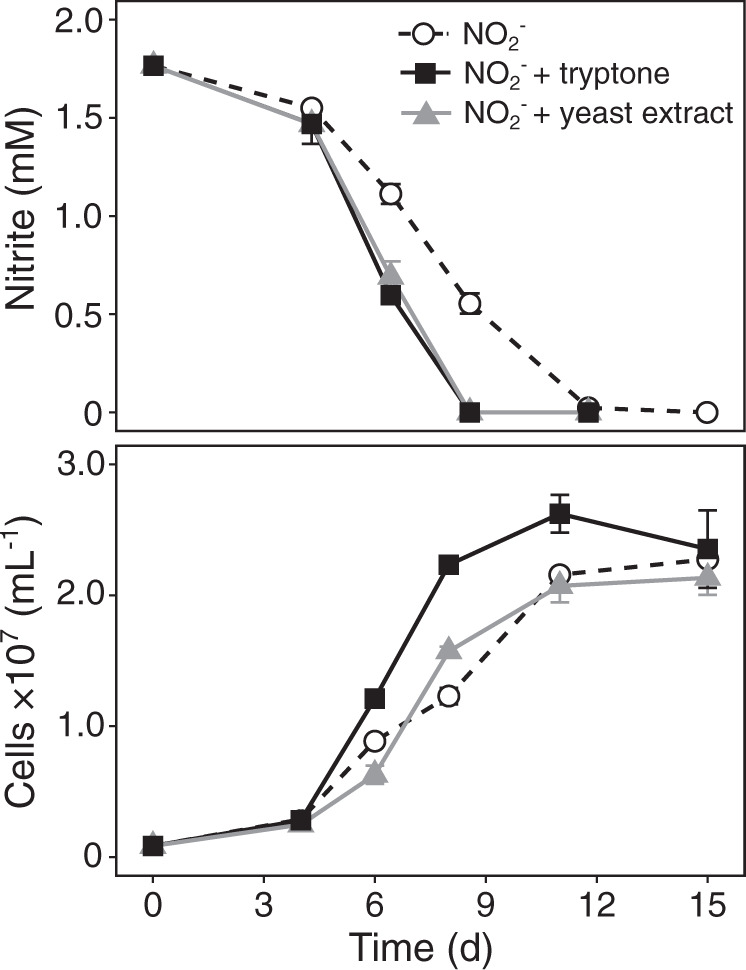
The effect of undefined organic carbon substrates on nitrite oxidation and growth of *N*. *marina* Nb-295^T^. Error bars represent the range of measurements from duplicate cultures.

**Fig. 3:**
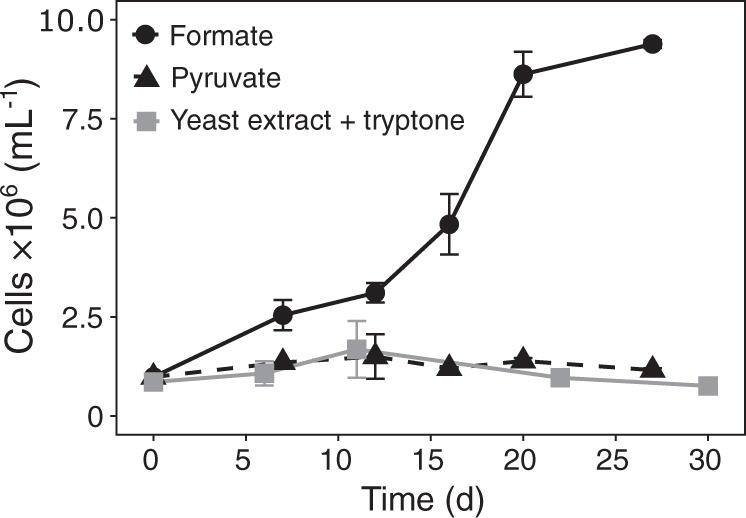
Growth of *N. marina* Nb-295^T^ on formate (1 mM), pyruvate (1 mM) and yeast extract and tryptone (150 mg L^−1^ each) in the absence of nitrite. Error bars represent the range of measurements from duplicate cultures.

In agreement with Watson et al. [5], NO_2_^−^ oxidation activity was enhanced when undefined organic compound mixtures such as tryptone and yeast extract were added to the culture medium (Fig. 2). Interestingly, growth was greatly stimulated by tryptone (Fig. 2), while amendment with defined organic carbon compounds had no effect on NO_2_^−^ oxidation or growth (Table S3). Parallel incubations with ammonium as an N source did not increase activity or growth (Table S3), suggesting that the stimulating effect of tryptone, and to a lesser extent of yeast extract, most likely can be attributed to direct amino acid assimilation and does not only reflect the eliminated energy demand for assimilatory NO_2_^−^ reduction. No growth on yeast extract and tryptone was observed in the absence of NO_2_^−^ (Fig. 3), corroborating their use as source of amino acids rather than for energy conservation.

In addition to undefined organic carbon substrates, defined organic compounds such as glycerol and pyruvate have been reported to enhance the growth of *N. marina* and *N. defluvii*, respectively [5, 12]. Formate has also been shown to serve as an electron donor and carbon source for some lineage I and II *Nitrospira* [11, 28]*. N. marina* encodes a putative formate dehydrogenase (FdhA) (Data Set 1), which is divergent from those found in *N. moscoviensis* and *N. defluvii* (∼24 and 27% amino acid identity, respectively), but shares a relatively high sequence similarity (∼48% amino acid identity) to the functionally characterized formate dehydrogenase Fdh4 from *Methylobacterium extorquens* [66]. *N. marina* was able to grow chemoorganotrophically on 1 mM formate in the absence of NO_2_^−^ (Fig. 3) and is thus not an obligate chemolithotrophic organism. However, the use of formate instead of NO_2_^−^ as electron donor resulted in slower growth rates, as previously also observed in *N. moscoviensis* [28]. In contrast to earlier reports [5], additions of 1 g L^−1^ glycerol did not stimulate NO_2_^−^ oxidation activity or growth (Table S3). Furthermore, pyruvate could neither be used as alternative energy source (Fig. 3), nor did it stimulate metabolic activity in the presence of NO_2_^−^ (Table S3).

#### Protection against oxidative, osmotic, and UV light-induced stress

The formation of reactive oxygen species is prevalent in oxic environments and oxidative stress defense is an important component of the stress response in marine organisms [67]. *N. marina* encodes multiple enzymes to reduce oxidative stress, including a cytoplasmic Mn/Fe-binding SOD, a periplasmic Cu/Zn-binding SOD, two heme-containing catalases, and various peroxiredoxins (Data Set 1). In contrast, *Nitrospina gracilis* and marine ammonia-oxidizing archaea lack catalase [48, 58], suggesting that *N. marina* is less susceptible to oxidative stress compared to other marine nitrifiers. In addition to its plethora of oxidative stress defense-related proteins, *N. marina* also encodes two putative photolyases—enzymes known to be involved in the repair of UV light-induced DNA damage [68]—suggesting that it is well adapted to conditions characteristic for euphotic environments.

Marine microorganisms counteract the external osmotic stress from high salt concentrations by accumulating a variety of organic solutes (=osmolytes) in the cytoplasm, which can either be synthesized de-novo or transported into the cell from the surrounding environment [69]. In addition to select amino acids that can serve as compatible solutes (e.g., proline and glutamate) [70], biosynthesis pathways for the osmolytes glycine betaine and trehalose were identified in the *N. marina* genome (see Supplementary Results and Discussion). The production and concomitant release of osmolytes (i.e., via diffusion, excretion, predation or cell lysis) could potentially fuel heterotrophic metabolism in the ocean [71] representing a link between chemolithoautotrophic production and heterotrophic consumption of DOM as recently suggested for ammonia-oxidizing archaea [72].

#### Vitamin B_12_ auxotrophy

**Fig. 4:**
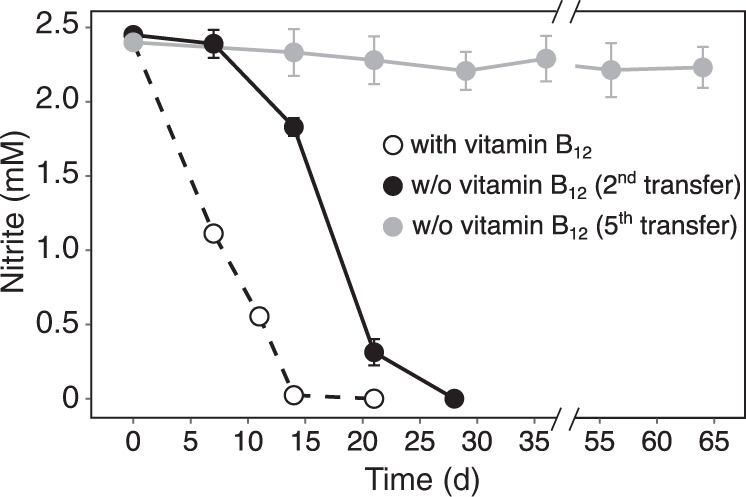
Nitrite consumption by *N. marina* Nb-295^T^ in artificial seawater mineral medium with and without addition of vitamin B_12_ (Table S1). Cultures of strain Nb-295^T^ were repeatedly transferred into medium without vitamin B_12_ using 2% inocula. Error bars represent the range of measurements from duplicate cultures.

B vitamins are important biochemical co-factors required for cellular metabolism and their concentrations are depleted to near zero across large areas of the global ocean [73]. *N. marina* encodes complete biosynthetic pathways for the B vitamins thiamin (B_1_), riboflavin (B_2_), pantothenate (B_5_), pyridoxine (B_6_), biotin (B_7_) and tetrahydrofolate (B_9_). However, an incomplete vitamin B_12_ biosynthesis pathway was identified (Fig. S2), lacking genes for multiple precorrin conversion reactions that ultimately lead to the biosynthesis of the molecule’s corrin ring [74]. Since *N. marina* only encodes the cobalamin-dependent versions of ribonucleotide reductase, methionine synthase and methylmalonyl-CoA mutase, it must rely on the supply of vitamin B_12_ or its precursors by other members of the microbial community. Indeed, nitrite consumption by *N. marina* ceased after repeated transfers into an artificial seawater mineral medium without the addition of vitamin B_12_ (Fig. 4), and nitrite oxidation activity was restored after adding vitamin B_12_ to B_12_-deplete cultures (data not shown). Interestingly, the *N. marina* genome contains genes for multiple reactions that convert the precursor cobyrinate/hydrogenobyrinate to cobalamin, and encodes all genes required for cobalamin salvage from cobinamide (Fig. S2, Data Set 1). Hence, in contrast to many other bacteria that lack the complete vitamin B_12_ biosynthesis pathway [75], *N. marina* appears to obtain its B_12_ from salvage of multiple intermediates from the environment. Vitamin B_12_ auxotrophy has recently also been observed for several marine Nitrospinae strains [76]. In contrast, Park et al. [77] suggested that the two novel marine NOB strains MSP and DJ, one of which is closely related to *N. marina*, might be able to synthesize cobalamin. However, given that these cultures were not axenic and that genes involved in precorrin conversion reactions absent in *N. marina* (Fig. S2) are also missing from their genomes, it is likely that both strains depend on the exogenous supply of vitamin B_12_ as well.

When *N. marina* was grown in natural seawater, cellular concentrations of ribonucleotide reductase were approximately 39-fold higher than for the cyanobacterium *Prochlorococcus* [78] (Table 1) despite only having a ~two-fold greater cell volume, consistent with the importance of B_12_ nutrition to *N. marina*. Moreover, this salvage acquisition mode is interesting in the context of dissolved cobalt speciation, which is complexed by strong organic ligands hypothesized to include B_12_ precursors or degradation products [79]. In the Northwest Atlantic near where *N. marina* was isolated, organic cobalt complexes are abundant, comprising about half the dissolved cobalt inventory [80].

**Table 1:** Targeted quantitative analyses of selected proteins using isotopically-labeled (^15^N) peptide standards.

Target protein^a^	^15^N-Peptide sequence	Peptide concentration (copies cell ^−1^)	
		atm. O_2_	low O_2_
Nitrite oxidoreductase, alpha subunit 1	LVVITPEYNPTAYR	845 (52)	1924 (275)
	DYAFPDFANSYSGK	241 (40)	451 (91)
	HPFWEETNESKPQWTR	673 (39)	1571 (388)
Nitrite oxidoreductase, alpha subunit 2	VVVITPEYNPTAQR	1,154 (27)	851 (187)
Nitrite oxidoreductase, alpha subunit 3	DYQFPDFTSTYSGK	1,761 (159)	1,261 (242)
	SGIDPALTGTHR	4,519 (516)	3,400 (526)
	IAVITPEYNPTAYR	4,860 (496)	3,741 (710)
Nitrite oxidoreductase, alpha subunit (all)	GWKPSDPYYK	11,467 (1,957)	9,564 (1,083)
	AIALDTGYQSNFR	13,996 (962)	13,116 (2,934)
Pyruvate:ferredoxin oxidoreductase,	TPSFFTGSEVIK	2,026 (192)	1,550 (248)
alpha subunit	EAIAILEEEGIR	1,362 (80)	1,054 (170)
	EVSATVPNNER	2,453 (169)	1,736 (215)
Ribonucleotide reductase	TGESPYQTIPFSHR	300 (46)	198 (30)
	EAAVPEPYIHR	705 (132)	472 (77)
	IINQSLPPALR	750 (88)	546 (68)

Nitrite oxidoreductase peptide targets were designed to match a specific NxrA copy (1, 2, or 3), or to match all three as indicated in the target protein column. Values represent the mean of triplicate measurements and standard deviations are shown in brackets.

^a^A complete list of quantified proteins can be found in Data Set 2.

### Metabolic response to low oxygen concentrations

Given the presence of multiple signatures of microaerophilic adaptation and metabolic diversity of nitrite oxidizers [26, 28, 58, 62] and their occurrence in low oxygen environments [25, 81, 82], we sought to further investigate potential adaptations of *Nitrospira marina* Nb-295^T^ to low O_2_ conditions. *N. marina* was grown at O_2_ concentrations characteristic for the upper ocean (∼200 µM) and at O_2_-limiting conditions (∼5.6 µM O_2_) found in environments with elevated NO_2_^−^ concentrations such as OMZ or sediments [83, 84].

**Fig. 5:**
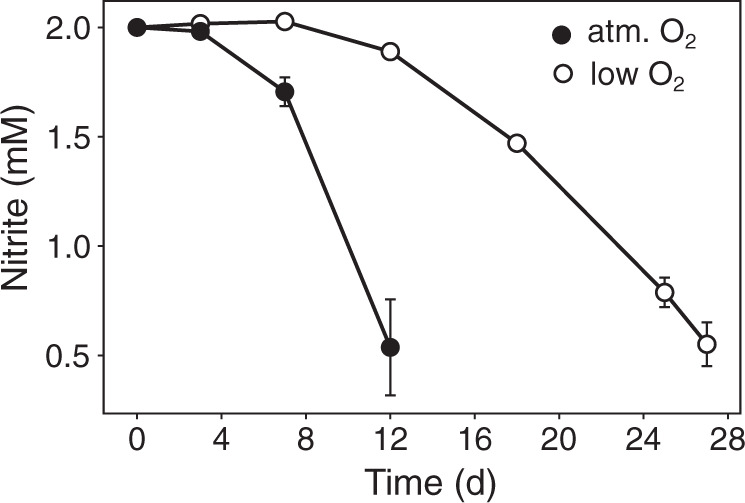
Nitrite consumption by *N. marina* Nb-295^T^ grown under atmospheric (filled circles) and low O_2_ conditions (open circles). Cells for proteome analysis were harvested after 12 and 27 days, respectively. Error bars represent standard deviations from measurements of triplicate cultures.

When grown under atmospheric O_2_ concentration, *N. marina* oxidized 1.5 mM NO_2_^−^ within 12 days, whereas under O_2_-limiting conditions depletion of 1.5 mM substrate took 27 days (Fig. 5). This result is in agreement with the initial description by Watson et al. [5], who reported partial, though unquantified, inhibition at low O_2_ partial pressure. Cell abundances at the final timepoint (after 1.5 mM NO_2_^−^ was oxidized) were comparable for both treatments (Fig. S3), indicating that the reduced NO_2_^−^ oxidation rate during O_2_-limiting conditions ultimately resulted in similar cell yields.

#### General proteomic response and upregulation of gene clusters

**Fig. 6:**
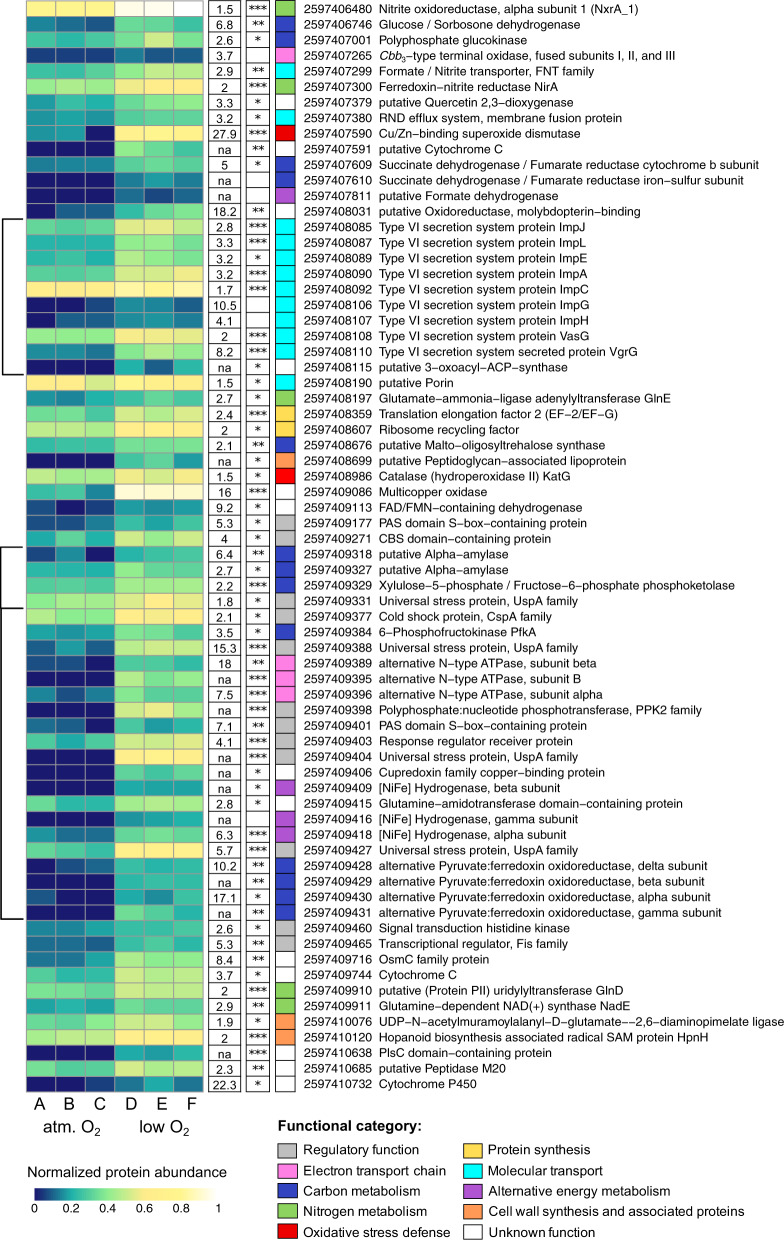
Heat map of *N. marina* Nb-295^T^ proteins that were more abundant under low O_2_ concentrations compared to atmospheric O_2_ concentration. Relative protein abundance values were square-root transformed and hypothetical proteins were excluded to improve readability. The complete set of untransformed values can be found in Data Set 3. Fold-changes and significance values (adj. *P* value ≤ 0.001, ***; ≤0.01, **; ≤0.05, *) are shown in white boxes next to the corresponding protein. Select low abundant proteins of interest with high fold-changes were included despite being not statistically significant (see “Material and Methods”). Fold-changes of proteins that were not detected under atmospheric O_2_ conditions are omitted to avoid dividing by zero (not available, na). Functional categories of depicted proteins are indicated by different colors. Gene clusters are indicated by black brackets.

Cultures grown under both O_2_ treatments were harvested for proteomic analysis when [NO_2_^−^] dropped to ~500 µM, corresponding to exponential growth of strain Nb-295 (see “Material and Methods”). A total of 2031 and 2046 proteins were identified by liquid chromatography-tandem mass spectrometry in the atmospheric and low O_2_ treatments, respectively, accounting for 48.1 and 48.5% (49.7% combined from a total of 175,653 peptides) of the predicted protein coding sequences in the *N. marina* genome. As previously reported for *N. marina* and *Nitrococcus mobilis*, proteins exhibiting the highest abundances were associated with NO_2_^−^ oxidation [44] (Data Set 1). NXR made up on average 4% of all peptide spectral counts and cellular NXR concentrations were ~13,500 copies cell^−1^ as determined by targeted quantitative proteomic analyses (Table 1), covering an estimated 35% of the membrane surface (see “Material and Methods”). All three NxrA copies were detected in the proteome as determined by detection of unique peptides of each, and NxrA_3 appeared to be more abundant compared to NxrA_1 and NxrA_2 (Table 1). Under low O_2_ conditions, NxrA_1 increased in abundance (Table 1, Fig. 6) indicating different metabolic or regulatory roles of the highly similar subunits.

Proteins involved in CO_2_ fixation, DNA replication, electron transport and central carbon metabolism were also highly abundant under both conditions, indicating that *N. marina* retained its central metabolism during O_2_ deficiency (Data Set 1). Although proteomic spectral counts remained constant for the majority of proteins during both treatments, spectral counts of 93 proteins significantly increased (adjusted *P* value ≤ 0.05) during growth at low O_2_ concentrations (Fig. 6, Data Set 3). These results are supported by the targeted quantitative proteomic analyses (Fig. S4, Data Set 2), suggesting that spectral counts are a good proxy for changes in absolute protein abundance in our dataset.

Multiple universal stress proteins (UspA superfamily) were among the proteins that showed the highest increase in abundance under low O_2_ conditions compared to the control treatment (Fig. 6, Data Set 3). UspA proteins have versatile regulatory and protective functions to enable survival under diverse external stresses [85], and are induced during growth inhibition [86] and oxygen starvation [87]. In *N. marina*, all four upregulated UspA proteins are located upstream or downstream of operons containing genes that also increased in abundance under low O_2_ conditions (Fig. 6), suggesting a regulatory role of UspA-related proteins upon O_2_ limitation. These upregulated gene clusters include proteins involved in electron transport, carbon metabolism, and alternative energy metabolism (Fig. 6).

In addition to putative UspA-regulated gene clusters, a gene cluster containing type VI secretion system (T6SS)-related proteins exhibited higher abundances under low O_2_ conditions (Fig. 6). The T6SS is typically involved in the secretion of effectors required for pathogenesis, bacterial competition, biofilm formation, and cell communication (e.g., quorum sensing) [88, 89]. While quorum sensing has recently been shown for diverse NOB including *N. moscoviensis* [90], no LuxI autoinducer synthases and/or LuxR signal receptor homologs were identified in the *N. marina* genome.

#### Induction of a putative O_2_-sensitive 2-oxoacid:ferredoxin oxidoreductase complex

The key enzymes of the rTCA cycle, pyruvate:ferredoxin oxidoreductase (POR) and 2-oxoglutarate:ferredoxin oxidoreductase (OGOR), are typically highly O_2_ sensitive because they contain easily oxidized iron-sulfur clusters [91]. In *Hydrogenobacter thermophilus*, five-subunit O_2_-tolerant forms of POR and OGOR mainly support aerobic growth, while a O_2_-sensitive two-subunit form is used under anaerobic conditions [92]. *N. marina* encodes three 2-oxoacid:ferredoxin oxidoreductase gene clusters that could exhibit POR or OGOR activity (Data Set 1). Two of these gene clusters consist of five CDS that exhibit a high sequence similarity to the O_2_-tolerant five-subunit POR/OGOR of *H. thermophilus* [93, 94], as previously described for *N. defluvii* [26]. Both complexes were highly abundant in *N. marina* proteomes from atmospheric and low O_2_ treatments (Data Set 1, Table 1) confirming their important role in central carbon metabolism. The third cluster contains alpha, beta, and gamma subunits of a putative POR with homology to the functionally characterized four-subunit PORs of the anaerobic thermophiles *Pyrococcus* and *Thermotoga* [95] and is absent in all other *Nitrospira* with the exception of *Ca*. N. alkalitolerans [9]. A protein with a 4Fe-4S binding domain was identified in the same operon, potentially representing the missing delta subunit of the POR complex (Data Set 1). This putative four-subunit POR was among the proteins that showed the highest increase in abundance under low O_2_ conditions in *N. marina* (Fig. 6). The O_2_-tolerant POR/OGOR isoforms were reported to have a >5-times lower specific activity [92] and might therefore constitute a substantial part of the cellular soluble protein content in *H. thermophilus* [96]. Hence, it is tempting to speculate that *N. marina* increases the expression of a more efficient (i.e., higher specific activity), O_2_-sensitive four-subunit POR under O_2_ limitation. While oxidative stress typically decreases under low O_2_ conditions, it might still be high enough to damage O_2_-sensitive enzymes. In *N. marina*, the abundance of a periplasmic Cu/Zn-binding SOD and a cytoplasmic catalase (KatG) increased under O_2_-limited conditions (Fig. 6), while the abundances of other oxidative stress defense-related proteins remained constant (Data Set 1 and 3). SOD has been shown to be efficient in protecting POR activity from oxidative damage in *Entamoeba histolytica* [97], and was among the proteins with the highest increase in abundance under low O_2_ conditions in *N. marina* (fold-change: 27.9), suggesting a role in POR protection.

#### Expression of a high O_2_-affinity *cbb*_*3*_-type terminal oxidase

The majority of proteins related to electron transport showed similar abundance levels at atmospheric and limiting O_2_ concentrations (Data Set 1 and 3). Despite its overall low abundance, a putative high-affinity cytochrome *cbb*_*3*_-type terminal oxidase was 3.6-times more abundant under low O_2_ concentrations compared to atmospheric O_2_ tension (Fig. 6). The *cbb*_*3*_-type terminal oxidase of ﻿*Bradyrhizobium japonicum* was reported to have a *K*_m_ value of 7 nmol L^−1^ O_2_ [98] and NO_2_^−^ oxidation rates have been detected at O_2_ concentrations in the low nanomolar range (5–33 nmol L^−1^ O_2_) ﻿[25]. This suggests that the *cbb*_*3*_-type terminal oxidase might enable *N. marina* to continue aerobic respiration at low O_2_ concentrations, albeit at lower NO_2_^−^ oxidation rates (Fig. 5). ﻿Low-affinity terminal oxidases are typically more efficient in energy conservation [99], suggesting that *N. marina* benefits from the presence of a terminal oxidase with lower O_2_ affinity in well-oxygenated environments. While *N. gracilis* encodes a highly similar high affinity *cbb*_*3*_-type terminal oxidase to *N. marina* [58], this enzyme is lacking in other Nitrospinae, including those identified in OMZs and sediments [76, 82, 100]. Interestingly, these genomes also lack the putative terminal oxidase proposed for *Nitrospira* [26]. Still, in an OMZ where Nitrospinae bacteria were the only detected NOB, NO_2_^−^ oxidation rates already approached saturation at ∼1 μmol L^−1^ O_2_ [25], indicating that Nitrospinae bacteria might be better adapted to low O_2_ concentrations compared to *N. marina*, but the enzyme conferring high O_2_ affinity in Nitrospinae remains to be identified.

#### Increase in abundance of proteins involved in alternative energy metabolism

The abundance of proteins putatively involved in alternative energy metabolisms increased under low O_2_ concentrations. These included Group 3b [NiFe] hydrogenase and formate dehydrogenase (Fig. 6). Alternative electron donors such as H_2_ and formate are common reaction products at oxic/anoxic interfaces [101]. *N. moscoviensis* can couple H_2_ and formate oxidation to NO_3_^−^ reduction to remain active under anoxia [28, 30] (whereas no net growth was observed for the former [30]), however, it is unlikely that H_2_ or formate were present at the culture conditions in this study. While abundances of hydrogenase and formate dehydrogenase increased under low O_2_ conditions, they were overall still comparably low (Fig. 6), suggesting that their expression might be upregulated when conditions become more unfavorable for aerobic NO_2_^–^ oxidation. Curiously, multiple subunits of the Na^+^-translocating N-type ATPase exhibited higher abundances at low O_2_ concentration (Fig. 6). Na^+^-translocating ATPases are suggested to be ancient enzymes that were later replaced by energetically more favorable H^+^-translocating ATPases [102]. While only few obligate anaerobes with very tight energy budgets (which cannot cover the losses caused by proton leaks) primarily use Na^+^ energetics, many organisms retained Na^+^ pumps and utilize them under energetically less favorable conditions such as anaerobiosis [102]. Hence, expression of a Na^+^-translocating ATPase suggests an adaptation of *N. marina* to overcome periods of starvation when energetically favorable electron donors or acceptors are short in supply.

## Conclusions

Although the vast majority of the ocean is well oxygenated, oxygen-depleted zones exist within the oceanic water column and in marine sediments [83, 84] with consequences for microbial adaptation and evolution. Our results show that, in contrast to Nitrospinae-dominated NOB populations in low oxygen waters [25], NO_2_^−^ oxidation activity of *Nitrospira marina* was reduced when grown at ∼5.6 µM O_2_, suggesting different O_2_ adaptations among different marine NOB. We confirm that *N. marina* benefits from the addition of undefined organic carbon substrates, which were shown to be inhibitory for *Nitrospina gracilis* [103], potentially further contributing to ecological niche partitioning within marine NOB. Our results indicate that *N. marina* is highly metabolically versatile, which might enable it to survive under unfavorable conditions with fluctuating levels of electron donors and acceptors. Hence, while Nitrospinae bacteria are the dominant nitrite oxidizers in oligotrophic oceanic regions and OMZs, *Nitrospira* might be better adapted to well-oxygenated high productivity regions including coastal systems, deep-sea trenches and hydrothermal vents. Finally, our results also indicate several ways that NOB may interact with other members of the marine microbial community—through the supply of organic carbon-containing osmolytes and their requirement for exogenous vitamin B_12_.

## Supplementary information

Supplementary Information

Data Set 1

Data Set 2

Data Set 3

## Data Availability

The genome of *Nitrospira marina* Nb-295^T^ is available in the JGI IMG/M repository under genome ID number 2596583682. Manually curated protein annotations are available in Data Set 1, targeted quantitative proteomic analyses results are available in Data Set 2, global proteomic spectral counts and differential expression analysis results are available in Data Set 3. Raw mass spectra are available in PRIDE as project number PXD021606. Data is archived at Biological and Chemical Oceanography Data Management office (BCO-DMO) under project 806565.
