# Supplementary material for: Metabolic versatility of the nitrite-oxidizing bacterium Nitrospira marina and its proteomic response to oxygen-limited conditions
Source: ISME J. 2020 Nov 23;15(4):1025–39. doi: 10.1038/s41396-020-00828-3 (PMC8115632; doi:10.1038/s41396-020-00828-3)
Supplement: Supplementary file 1 — Supplementary Information [file 41396_2020_828_MOESM1_ESM.pdf]

Supplementary Information (SI)

Supplementary Methods

Nitrite measurements

NO_2^- concentrations were measured using the Griess-Ilosvay colorimetric method [1]. Briefly, samples were diluted 1:100 with MilliQ water, and 2 mL were transferred to plastic cuvettes, to which 100 µL of sulfanilamide (10 g L^{-1} in 10% HCl) and N-(1-naphthyl)ethylenediamine (1 g L^{-1}) were added. Cuvettes were shaken, incubated for 5 min in the dark and absorbance was measured at 545 nm on a Thermo Scientific Evolution 60S UV-Visible spectrophotometer. Standards (0-2.5 μM NO_2^-) were run in parallel and then used to calculate the concentration in each sample.

Flow cytometry cell counts

Samples of 500 µL were collected in 2 mL sterile polypropylene tubes, fixed with glutaraldehyde (0.5% final concentration) for 10 min and subsequently frozen and stored at -80°C. Prior to analysis, samples were diluted (1:10, 1:50 or 1:100, depending on the cell concentration) in 0.2 µm filtered Tris-EDTA buffer (1M Tris-HCl, 0.1M EDTA, pH 8) and stained with SYBR Green I (Invitrogen) at a final concentration of 1:5,000 for 30 min. Enumeration of cells was performed on an Easy-Cyte flow cytometer (Guava Technologies).

Protein extraction and purification

Samples were resuspended with 1800 μL of 1% SDS extraction buffer (1% SDS, 0.1M Tris/HCl pH 7.5, 10mM EDTA). Each sample was incubated at room temperature for 15 min, heated at 95°C for 10 min, and shaken at room temperature (RT) at 350 rpm for 1 h. The protein extracts were decanted and centrifuged at 14100 x g for 20 min at RT. The supernatants were removed and concentrated by membrane centrifugation to approximately 300 μL in 5 K MWCO Vivaspin units (Sartorius Stedim, Goettingen, Germany). Each sample was precipitated with cold 50% methanol/50% acetone/0.5 mM HCl for 3 days at –20°C, centrifuged at 14100 x g for 30 min at 4°C, decanted and dried by vacuum concentration (Thermo Savant Speedvac) for 10 min or until dry. Pellets were resuspended in 1% SDS extraction buffer and left at RT for 1 h to completely

dissolve. Total protein was quantified (Bio-Rad DC protein assay, Hercules, CA) with BSA as a standard.

Extracted proteins were purified from SDS detergent, reduced, alkylated and trypsin digested while embedded within a polyacrylamide tube gel, modified from a previously published method [2]. A gel premix was made by combining 1 M Tris HCl (pH 7.5) and 40% Bis-acrylimide L 29:1 (Acros Organics) at a ratio of 1:3. The premix (103 μL) was combined with an extracted protein sample (35 μg-50 μg), TE Buffer, 7 μL 1% APS and 3 μL of TEMED (Acros Organics) to a final volume of 200 μL. After 1 h of polymerization at RT, 200 μL of gel fix solution (50% ETOH, 10% acetic acid in LC/MS grade water) was added to the top of the gel and incubated at RT for 20 min. Liquid was then removed and the tube gel was transferred into a new 1.5 mL microtube containing 1.2 mL of gel fix solution then incubated at RT, 350 rpm in a Thermomixer R (Eppendorf) for 1 h. The gel fix solution was then removed and replaced with 1.2 mL destaining solution (50% MeOH, 10% acetic acid in H_2O) and incubated at 350 rpm for 2 h. Liquid was then removed, gel cut up into 1 mm cubes and then added back to tubes containing 1 mL of 50:50 acetonitrile:25 mM ammonium bicarbonate (ambic) incubated for 1 h, 350 rpm at RT. Liquid was removed and replaced with fresh 50:50 acetonitrile:ambic and incubated at 16°C at 350 rpm overnight. The above step was repeated for 1 h the following morning. Gel pieces were then dehydrated twice in 800 μL of acetonitrile for 10 min at room temperature and dried for 10 min in a ThermoSavant DNA110 speedvac after removing solvent. 600 μL of 10 mM DTT in 25 mM ambic was added to reduce proteins incubating at 56°C, 350 rpm for 1 h. Unabsorbed DTT solution was then removed with volume measured. Gel pieces were washed with 25 mM ambic and 600 μL of 55 mM iodacetamide was added to alkylate proteins at RT, 350 rpm for 1 h. Gel cubes were then washed with 1 mL ambic for 20 min, 350 rpm at RT. Acetonitrile dehydrations and speedvac drying were repeated as above. Trypsin (Promega #V5280) was added in appropriate volume of 25 mM ambic to rehydrate and submerse gel pieces at a concentration of 1:20 μg trypsin:protein. Proteins were digested overnight at 350 rpm and 37°C. Unabsorbed solution was removed and transferred to a new tube. 50 μL of peptide extraction buffer (50% acetonitrile, 5% formic acid in water) was added to gels, incubated for 20 min at RT then centrifuged at 14,100 x g for 2 min. Supernatant was collected and combined with unabsorbed solution. The above peptide extraction step was repeated combining all supernatants. Combined protein extracts were centrifuged at 14,100 x g for 20 min, supernatants transferred into a new tube and dehydrated down to approximately 10-20 μL in the speedvac. Concentrated peptides

were then diluted in 2% acetonitrile 0.1% formic acid in water for storage until analysis. All water used in the tube gel digestion protocol was LC/MS grade, and all plastic microtubes were ethanol rinsed and dried prior to use.

Global and targeted proteomic data analyses

Global proteomic data was produced using a Dionex Ultimate nanoLC system coupled to a Thermo Fusion mass spectrometer with a Thermo Flex ion source. 1 μg of each sample (measured before trypsin digestion) was concentrated onto a trap column (0.2 x 10 mm ID, 5 μm particle size, 120 Å pore size, C18 Reprosil-Gold, Dr. Maisch GmbH) and rinsed with 100 μL 0.1% formic acid, 2% acetonitrile (ACN), 97.9% water before gradient elution through a reverse phase C18 column (0.1 x 250 mm ID, 3 μm particle size, 120 Å pore size, C18 Reprosil-Gold, Dr. Maisch GmbH) at a flow rate of 500 nL min^{-1}. The chromatography consisted of a nonlinear 220 min gradient from 5% to 95% buffer B, where A was 0.1% formic acid in water and B was 0.1% formic acid in ACN (all solvents were Fisher Optima grade). The mass spectrometer was set to perform MS scans on the orbitrap (240000 resolution at 200 m/z) with a scan range of 380 m/z to 1580 m/z. MS/MS was performed on the ion trap using data-dependent settings (top speed, dynamic exclusion 15 seconds, excluding unassigned and singly charged ions, precursor mass tolerance of ±3ppm, with a maximum injection time of 50 ms). Global proteomic protein identifications were applied to data-dependent acquisition mode raw files using the SEQUEST peptide to spectrum mapping algorithm in Proteome Discoverer version 2.1 using trypsin enzyme digestion allowing 2 missed cleavages, with fragment tolerance of 0.6 Da and parent tolerance of 10.0 ppm, allowing fixed modification of +57 for carbamidomethyl on cysteine and +16 for methionine oxidation. Spectral counts were calculated in Scaffold's Proteome Software Version 4.10 using peptide false discovery rate of 0.2% and protein false discovery rate of 4.6%. For the DESeq2 analysis, total exclusive spectral counts were used (see Material and Methods in the main text). Normalized spectral counts (normalized to total spectra within each sample) and normalized spectral abundance factor (NSAF) (normalized by amino acid length in order to take into account protein size) are reported in Data Set 1 and Data Set 3, respectively. NSAF was calculated as follows:

$$NSAF_k = \left(\frac{PSM}{L}\right)_k / \sum_{i=1}^{N} \left(\frac{PSM}{L}\right)_i$$

in which the total number of spectral counts for the matching peptides from protein *k* (PSM) was divided by the protein length (*L*), and then divided by the sum of PSM/*L* for all *N* proteins.

When a FDR of <1% was applied to the protein results, the proteins detected with a mean spectral count ≥ 6 across all treatments used in the DESeq2 analysis were identical in each case, with the exception of 52 proteins, none of which changed in abundance in response to low O_2 concentrations. As a result, we interpret the results to be very similar if not equivalent to FDR <1% on the protein level, and protein IDs were also very similar with 2096 and 1993 detected proteins for FDR <5% and <1%, respectively.

Targeted analysis was conducted by mixing stable isotope labelled peptide standards that were produced using a plasmid inserted in *E. coli* containing the peptides of interest and calibrated against Pierce standard peptides (Pierce peptide calibration mixture 88321) and run in parallel reaction monitoring (PRM) mode on the Thermo Fusion mass spectrometer. Samples were injected onto a trap column (300 µm × 5 mm, 5-µm bead size, 100-Å pore size, C18 PepMap100, Thermo Scientific) at 10 µL min^{-1} and washed for 10 min with 0.1% formic acid in 2% acetonitrile. The trapped sample was then eluted at 500 nL min^{-1} onto a C18 column (100 µm × 150 mm, 3-µm particle size, 120-Å pore size, C18 Reprosil Gold, Dr. Maisch, packed in a New Objective PicoFrit column) with a 70-min nonlinear gradient (0.1% formic acid in water and 0.1% formic acid in 99.9% acetonitrile) into the mass spectrometer. PRM settings in the Fusion were set to 240,000 Orbitrap resolution at a scan range of 350–1,200 m/z for MS1, and a targeted mass list in the ion trap with collision-induced dissociation activation in normal scan rate mode with a maximum injection time of 35 ms for MS2 scans. PRM data was analyzed using Skyline (Skyline targeted mass spec software 16.2)[3].

Phylogenetic analyses

Phylogenomic analysis was performed using 120 concatenated phylogenetic marker genes of representatives of the phylum Nitrospirae/Nitrospirota as implemented in the Genome Taxonomy Database Toolkit (GTDB-tk) version 1.1.1. [4]. The multiple sequence alignment contained 95 genomes and metagenome-assemble genomes (MAGs) from the Genome Taxonomy Database (GTDB) (Release 04-RS89, 19th June 2019) [5], the genome of *Nitrospira marina* Nb-295^T (this study), the MAG of *Ca.* Nitrospira alkalitolerans [6] and two open ocean single-amplified genomes (SAGs) AC-738-G23 and AC-732-L14 [7]. All genomes were estimated to be ≥50% complete with ≤5% contamination based on CheckM [8]. Conserved marker genes were identified using the command 'gtdbtk identify' (default parameters) and aligned to the reference genome alignment using the command 'gtdbtk align' using a taxonomy filter for Nitrospirae/Nitrospirota (--taxa_filter p__Nitrospirota,p__Nitrospirota_A) as implemented in GTDB-Tk. The resulting alignment was

used to calculate a maximum likelihood phylogenetic tree with IQ-Tree version 1.6.9 [9] based on the best-fit model (LG+F+R8), with ultrafast bootstrap (UFBoot) inferred from 10,000 replicates. The phylogenetic tree was visualized with the online tool iTOL [10].

Full-length 16S rRNA gene sequences were obtained from the GenBank database at the National Center for Biotechnology Information (NCBI) [11], except for *Nitrospira marina* Nb-295^T and the SAGs AC-728-G23 and AC-728-O15, which were obtained from the Integrated Microbial Genomes and Microbiomes (IMG) platform [12], and the sequence of *Ca.* Nitrosospira alkalitolerans KS, which was obtained from the MicroScope platform [13]. Gene sequences were aligned with MAFFT (L-INS-I method) [14] and unreliable positions were filtered from the resulting alignments with BMGE [15] resulting in 1361 nucleotide positions in the final alignment (except for the partial sequence of *N. bockiana* which only contained 1095 nucleotide positions). The phylogenetic tree was calculated by maximum likelihood with IQ-Tree version 1.6.9 [9] based on the best-fit model (TIM3+F+I+G4), with ultrafast bootstrap (UFBoot) inferred from 10,000 replicates. The phylogenetic tree was visualized with the online tool iTOL [10]. *Leptospirillum ferrooxidans* (X86776) was used as outgroup.

Supplementary Results and Discussion

Osmolyte biosynthesis

N. marina is able to synthesize the osmolyte trehalose via two different pathways (either directly from maltose using trehalose synthase or from glycogen via maltodextrin and maltooligosyl-trehalose) and encodes two putative glycine betaine/osmolyte transport systems (ABC and BCCT family) to import exogenous glycine betaine (Data Set 1). Furthermore, despite their diverse roles and intracellular functions, polyamines can also contribute to osmotic stress protection [16, 17]. *N. marina* can synthesize the polyamines putrescine and spermidine, and encodes a putative polyamine ABC transport module (Data Set 1). Protection against hypo-osmotic shock in *N. marina* might also be conferred by one large- and seven small-conductance mechanosensitive ion channels, Na^+:H^+ antiporters and voltage-dependent K^+ and Na^+ channels (Data Set 1).

Metal acquisition

Low concentrations of iron limit ocean productivity in many parts of the global ocean [18, 19] and could limit NOB due to the high iron requirements of their respiratory chain [20, 21]. *N. marina* encodes multiple metal transport systems, including a molybdenum ABC transporter module, two

putative zinc transporters (ZIP family), a putative Fe^{2+}/Mn^{2+} transporter (VIT1/CCC1 family), two putative copper-transporting P-type ATPases, and a putative high-affinity nickel transport protein (Data Set 1), however, it lacks an ABC-type uptake system for Fe^{3+} present in other characterized *Nitrospira* species [22]. Furthermore, no known proteins associated with siderophore production were identified in the *N. marina* genome. However, the presence of multiple TonB-like receptors suggests the potential for the uptake of iron-bound-siderophores produced by other microbes. Additionally, the *N. marina* genome contains three putative bacterioferritin-related proteins for iron storage. Only one complete ABC-type cobalamin/Fe^{3+} siderophore uptake system was identified which is co-localized with cobalamin biosynthesis and salvage genes, indicating that it transports cobalamin. An additional periplasmic substrate binding protein of a putative ABC-type cobalamin/Fe^{3+}-siderophore transport system is located in proximity to iron uptake-related genes including a ferric uptake regulator (Fur) and a ferritin-like protein (Data Set 1). Additionally, some major facilitator superfamily-type permeases have been shown to promote bacterial iron-siderophore import from the periplasm into the cytoplasm [23]. Alternatively, iron might be released from the siderophore by a reduction process in the periplasm and subsequently imported into the cytoplasm by one of the divalent cation transporters or the putative VIT1/CCC1 family Fe^{2+}/Mn^{2+} transporter, which would enable re-utilization of intact siderophores [23].

Supplementary References

1. Strickland JDH, Parsons TR. A Partical Handbook of Seawater Analysis. *Fish Res Bd Can, Bull No 167* 1972; 167.
2. Lu X, Zhu H. Tube-gel digestion: A novel proteomic approach for high throughput analysis of membrane proteins. *Mol Cell Proteomics* 2005; 4: 1948–1958.
3. Pino LK, Searle BC, Bollinger JG, Nunn B, MacLean B, MacCoss MJ. The Skyline ecosystem: Informatics for quantitative mass spectrometry proteomics. *Mass Spectrom Rev* 2020; 39: 229–244.
4. Chaumeil P-A, Mussig AJ, Hugenholtz P, Parks DH. GTDB-Tk: a toolkit to classify genomes with the Genome Taxonomy Database. *Bioinformatics* 2019; 36: 1925–1927.
5. Parks DH, Chuvochina M, Chaumeil P-A, Rinke C, Mussig AJ, Hugenholtz P. A complete domain-to-species taxonomy for Bacteria and Archaea. *Nat Biotechnol* 2020; doi: https://doi.org/10.1038/s41587-020-0501-8.

6. Daebeler A, Kitzinger K, Koch H, Herbold CW, Steinfeder M, Schwarz J, et al. Exploring the upper pH limits of nitrite oxidation: diversity, ecophysiology, and adaptive traits of haloalkalitolerant Nitrospira. *ISME J* 2020; doi: 10.1038/s41396-020-0724-1.
7. Pachiadaki MG, Sintes E, Bergauer K, Brown JM, Record NR, Swan BK, et al. Major role of nitrite-oxidizing bacteria in dark ocean carbon fixation. *Science* 2017; 358: 1046–1051.
8. Parks DH, Imelfort M, Skennerton CT, Hugenholtz P, Tyson GW. CheckM: Assessing the quality of microbial genomes recovered from isolates, single cells, and metagenomes. *Genome Res* 2015; 25: 1043–1055.
9. Nguyen LT, Schmidt HA, Von Haeseler A, Minh BQ. IQ-TREE: A fast and effective stochastic algorithm for estimating maximum-likelihood phylogenies. *Mol Biol Evol* 2015; 32: 268–274.
10. Letunic I, Bork P. Interactive Tree of Life (iTOL) v4: Recent updates and new developments. *Nucleic Acids Res* 2019; 47: 256–259.
11. Benson DA, Cavanaugh M, Clark K, Karsch-Mizrachi I, Lipman DJ, Ostell J, et al. GenBank. *Nucleic Acids Res* 2013; 41: D36-42.
12. Markowitz VM, Chen I-MA, Palaniappan K, Chu K, Szeto E, Grechkin Y, et al. IMG: the Integrated Microbial Genomes database and comparative analysis system. *Nucleic Acids Res* 2012; 40: D115-22.
13. Vallenet D, Calteau A, Dubois M, Amours P, Bazin A, Beuvin M, et al. MicroScope: an integrated platform for the annotation and exploration of microbial gene functions through genomic, pangenomic and metabolic comparative analysis. *Nucleic Acids Res* 2020; 48: D579–D589.
14. Katoh K, Standley DM. MAFFT multiple sequence alignment software version 7: Improvements in performance and usability. *Mol Biol Evol* 2013; 30: 772–780.
15. Criscuolo A, Gribaldo S. BMGE (Block Mapping and Gathering with Entropy): A new software for selection of phylogenetic informative regions from multiple sequence alignments. *BMC Evol Biol* 2010; 10: 210.
16. Miller-Fleming L, Olin-Sandoval V, Campbell K, Ralser M. Remaining Mysteries of Molecular Biology: The Role of Polyamines in the Cell. *J Mol Biol* 2015; 427: 3389–3406.
17. Groppa MD, Benavides MP. Polyamines and abiotic stress: Recent advances. *Amino Acids* 2008; 34: 35–45.
18. Ducklow HW, Oliver JL, Jr WOS. The Role of Iron as a Limiting Nutrient for Marine Plankton Processes. *Hydrosphere* 2000; 2: 295–310.
19. Martin JH. Iron as a Limiting Factor in Oceanic Productivity. *Prim Product Biogeochem*

Cycles Sea 1992; 123–137.

20. Saito MA, Mcilvin MR, Moran DM, Santoro AE, Dupont CL, Rafter PA, et al. Abundant nitrite-oxidizing metalloenzymes in the mesopelagic zone of the tropical Pacific Ocean. *Nat Geosci* 2020; 13: 355–362.
21. Santoro AE, Sakamoto CM, Smith JM, Plant JN, Gehman AL, Worden AZ, et al. Measurements of nitrite production in and around the primary nitrite maximum in the central California Current. *Biogeosciences* 2013; 10: 7395–7410.
22. Lücker S, Wagner M, Maixner F, Pelletier E, Koch H, Vacherie B, et al. A Nitrospira metagenome illuminates the physiology and evolution of globally important nitrite-oxidizing bacteria. *Proc Natl Acad Sci U S A* 2010; 107: 13479–13484.
23. Schalk IJ, Guillon L. Fate of ferrisiderophores after import across bacterial outer membranes: Different iron release strategies are observed in the cytoplasm or periplasm depending on the siderophore pathways. *Amino Acids* 2013; 44: 1267–1277.

Supplementary Figures and Tables

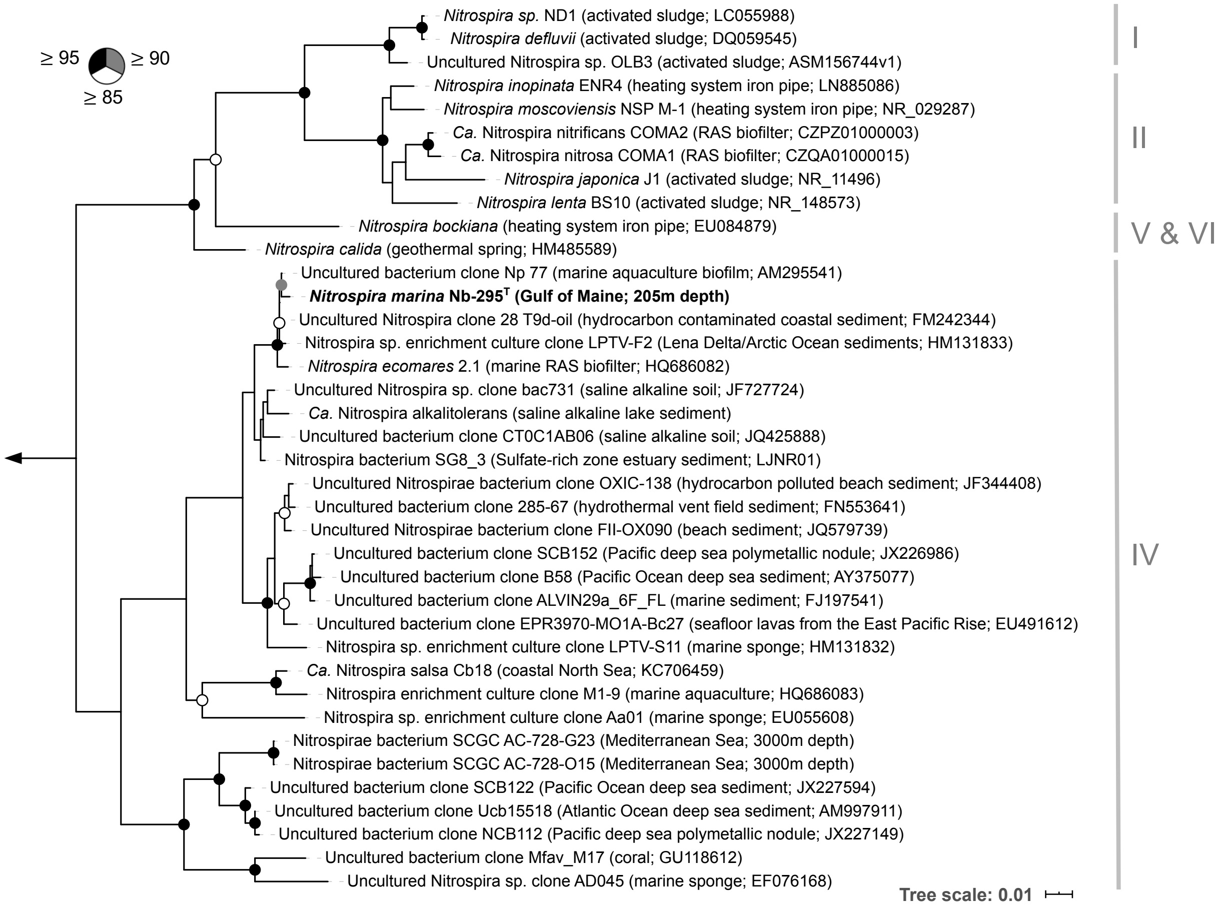

Fig S1. Maximum likelihood phylogenetic analysis of 16S rRNA gene sequences of cultured representatives and selected environmental sequences from the genus *Nitrospira*. *Leptospirillum ferrooxidans* (X86776) was used as outgroup indicated by the black arrow. Support values ≥85% are represented on the respective branches by circles color-coded as indicated in the figure. The scale bar represents 0.01 substitutions per site.

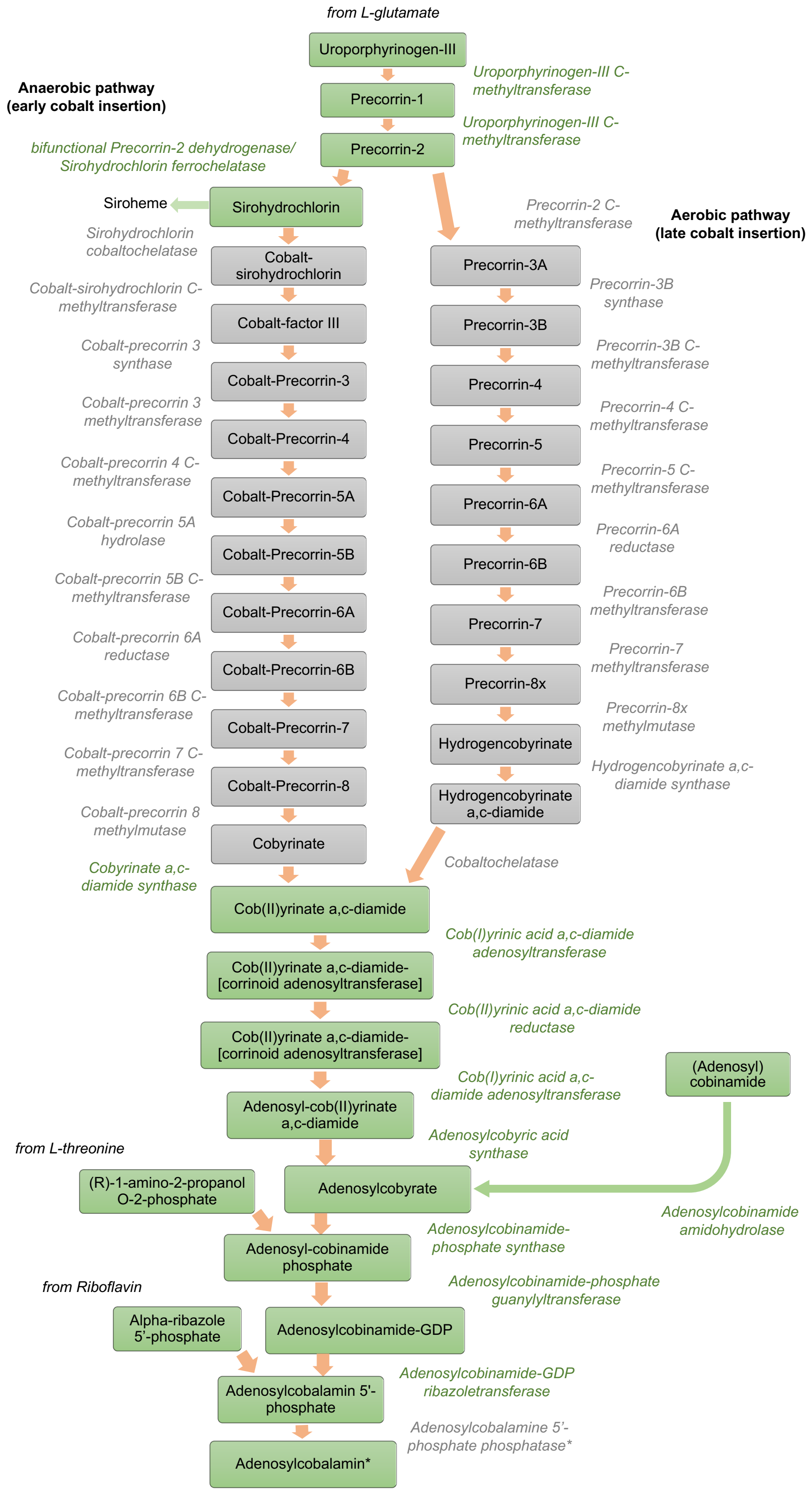

Fig S2. Depiction of the proposed incomplete vitamin B_{12} biosynthesis pathway in *N. marina* Nb-295^{T}, showing enzymes that are present (green) and absent (grey) from its complete genome sequence. *The last step of the pathway is likely carried out by an unidentified phosphatase.

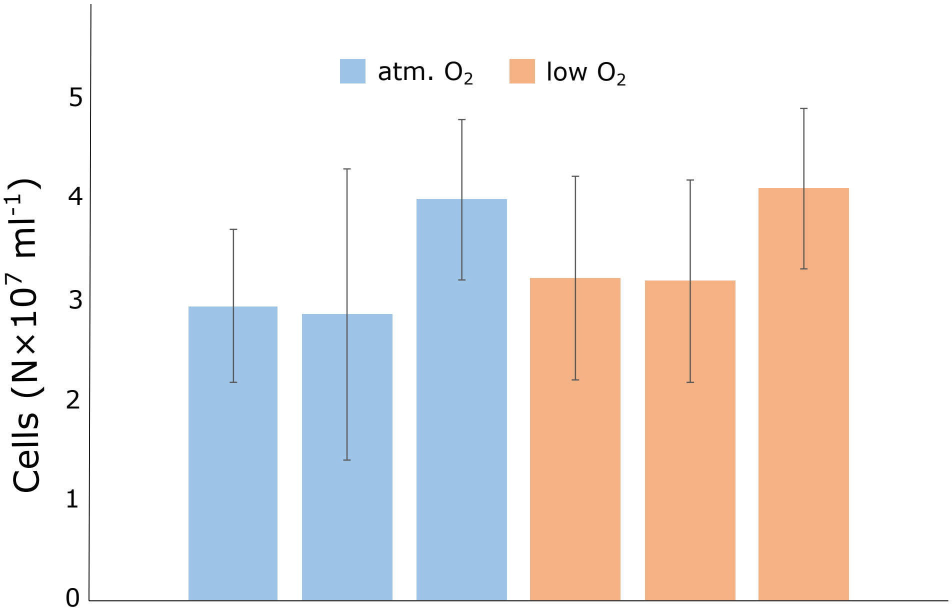

Figure S3. Cell counts of *N. marina* Nb-295 at the final time point of the oxygen experiments before cells were harvested for proteome analyses. Error bars represent standard deviations of cell counts from triplicate filters of the same sample.

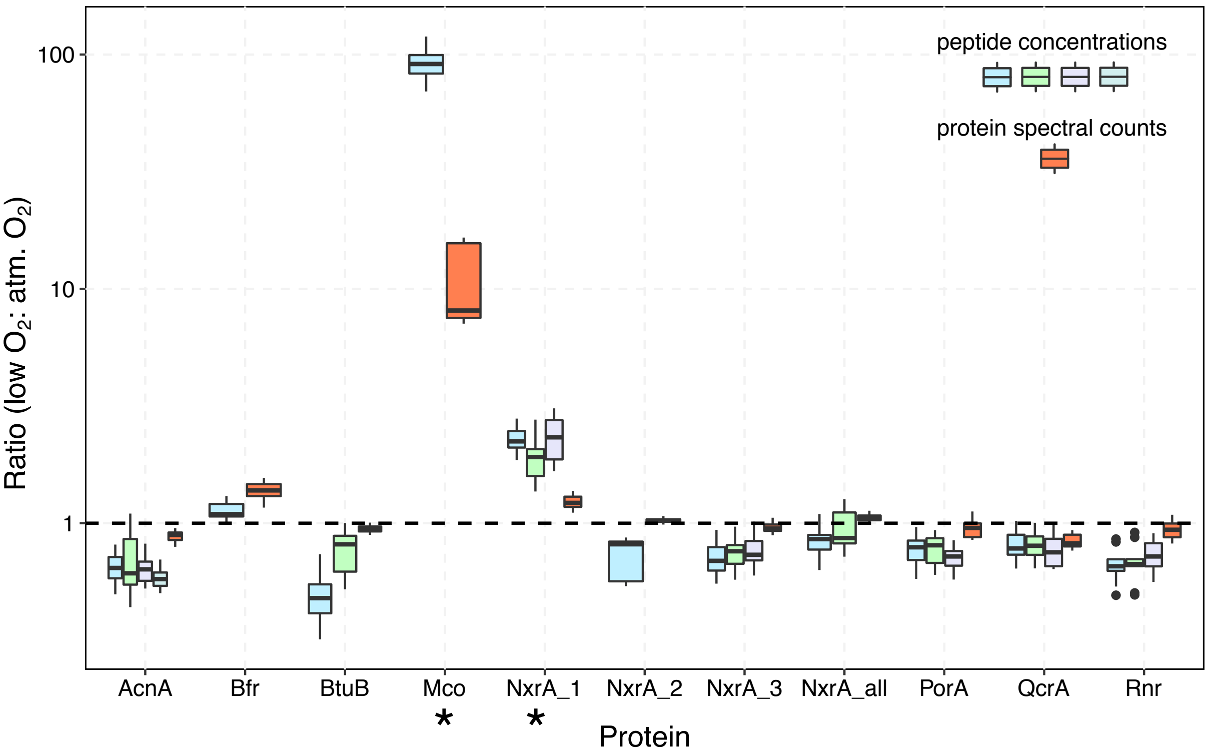

Figure S4. Comparison of proteomic spectral counts and quantified peptide concentrations. The dashed line indicates equal abundances in both treatments. Proteins marked with an asterisk (*) were significantly more abundant under low O_2 conditions (see Data Set 3 and Figure 6 in the main text).

Table S1. Composition of the nitrite oxidizer artificial seawater (NOASW) medium

Stock solutions:

**Basic salt solution**	**1L**	
NaCl	18.54	g
$MgSO_4\ 7H_2O$	4.70	g
$MgCl_2\ 6H_2O$	3.55	g
$CaCl_2\ 2H_2O$	1.03	g
KCl	0.51	g
$NaHCO_3$	0.14	g
fill up to 1L with MilliQ		

**Sodium nitrite solution (1M)**	**100**	**mL**
$NaNO_2$	6.9	g
fill up to 100mL with MilliQ		

**K_2HPO_4 solution (0.05M)**	**100**	**mL**
K_2HPO_4	0.87	g
fill up to 100mL with MilliQ		

**FeNaEDTA Solution (1g/L)**	**100**	**mL**
FeNaEDTA	100	mg
fill up to 100mL with MilliQ		

**Modified Non-chelated trace element mixture**			**1L**
Distilled H_2O	987	mL	
HCl (conc. ~12.5M)	8	mL	(100mM)
H_3BO_3	30	mg	(0.5mM)
$MnCl_2\ 4H_2O$	20	mg	(0.1mM)
$CoCl_2\ 6H_2O$	100	mg	(0.5mM)
$NiCl_2\ 6H_2O$	24	mg	(0.1mM)
$CuCl_2\ 2H_2O$	20	mg	(0.1mM)
$ZnSO_4\ 7H_2O$	144	mg	(0.5mM)
$Na_2MoO_4\ 2H_2O$	24	mg	(0.1mM)

**1000x Vitamin B12 solution (1mg/L)**	**1**	**L**
Cyanocobalamin	1	mg
fill up to 1L with MilliQ		

Medium preparation:

	1L	
Basic salt solution	995	mL
$NaNO_2$ (1M)	2	mL
Modified Trace Elements	1	mL
FeNaEDTA Solution (1 g/L)	0.5	mL
KH_2PO_4 (0.05 M)	0.5	mL
1000x Vitamin B12 solution	1	mL

Table S2. Genome characteristics of *N. marina* Nb-295 and other cultured nitrite-oxidizing bacteria

Nitrospira Lineage	IV	IV	I	II	II	II	II	II	II		
**Species**	***N. marina* Nb-295**	***Ca.* N. alkalitolerans KS**	**N. defluvii**	***N. moscoviensis* NSP M-1**	***N. japonica* NJ1**	***N. lenta* BS10**	***N. inopinata* ENR4**	***Ca.* N. nitrosa COMA1**	***Ca.* N. nitrificans COMA2**	***Nitrococcus mobilis* Nb-231**	***Nitrospina gracilis* 3/211**
Reference	this study	Daebeler et al. 2020	Lücker et al. 2010	Koch et al. 2014	Ushiki et al. 2018	Sakoula et al. 2018	Daims et al. 2015	van Kessel et al. 2015	van Kessel et al. 2015	Füssel et al. 2017	Lücker et al. 2013
Environment	ocean	saline alkaline lake	WWTP	heating system pipe	WWTP	WWTP	heating system pipe	WWTP	WWTP	ocean	ocean
Genome size (Mb)	4.68	4.94	4.32	4.59	4.08	3.76	3.30	4.42	4.12	3.62	3.07
Closed genome?	yes	no	yes	yes	yes	no	yes	no	no	no	no
Coding DNA sequences	4272	5091	4274	4508	4150	3968	3024	4309	4502	4052	3064
Average G+C content (%)	50	51.4	59	62	59	57.9	59.2	54.8	56.6	60	56.2
Number of rRNAs	3	3	3	4	3	3	3	3	3	3	3
Number of tRNAs	47	47	46	47	45	46	47	46	43	45	45
NxrAB operons	3	1[a].	2	4 (5)[b]	3	2	1	2	4	1[c]	2
Urease	–	–	–	+	+	+	+	+	+	–	–
Cyanase	+	+	+	+	+	+	–	–	–	+	+
Hydrogenase	3b	2a, 3b	–	2a	–	–	3b	3b	3b	3b	3b
Formate dehydrogenase	+	+	+	+	–	–	–	–	–	+	–
Catalase	2	1	0	3	2	0	1	0	1	1	0
Superoxide dismutase	2	2	0	1	2	2	0	0	1	2	0
Flagella	+	+	+	+	–	+	+	+	+	+	+

[a] contains one complete NxrAB operon, an additional complete NxrA copy and one fragmented NxrA copy at the end of a contig
[b] lost one NxrAB operon during prolonged cultivation
[c] contains an additional NxrA copy

Table S3. The effect of culture medium amendments on nitrite oxidation (A) and growth (B) of *N. marina* Nb-295. All incubations contained 2 mM $NaNO_2^-$ and 1X Vitamin B_{12} solution (see Table S1 for medium preparation). Nitrite concentrations are given in µM (A) and cell abundances in x10^5 ml^{-1} (B).

A

Time (d)	Control	Ammonia (100 µM)	Pyruvate (500 µM)	Glycerol (1 g L^{-1})	Yeast (150 mg L^{-1})	Tryptone (150 mg L^{-1})
0	1788 ± 2.8	1788 ± 2.8	1788 ± 2.8	1788 ± 2.8	1788 ± 2.8	1788 ± 2.8
7	519.5 ± 41.7	457 ± 1.41	507.5 ± 19.1	508 ± 114.6	60.5 ± 46	0 ± 0
11	0 ± 0	0 ± 0	0 ± 0	0 ± 0	0 ± 0	0 ± 0

B

Time (d)	Control	Ammonia (100 µM)	Pyruvate (500 µM)	Glycerol (1 g L^{-1})	Yeast (150 mg L^{-1})	Tryptone (150 mg L^{-1})
0	9.9 ± 0	9.9 ± 0	9.9 ± 0	9.9 ± 0	9.9 ± 0	9.9 ± 0
7	28.7 ± 0.7	26.5 ± 0.9	27.7 ± 2.1	30.3 ± 1	22.8 ± 0.7	38.5 ± 1.1
11	200.4 ± 15.2	202.8 ± 7.7	206.6 ± 9.1	206 ± 0.6	226.3 ± 36.6	253.2 ± 17.8
